# The behavioural preview effect with faces is susceptible to statistical regularities: Evidence for predictive processing across the saccade

**DOI:** 10.1038/s41598-020-79957-w

**Published:** 2021-01-13

**Authors:** Christoph Huber-Huber, David Melcher

**Affiliations:** 1 Center for Mind/Brain Sciences (CIMeC), University of Trento, Trento, Italy; 2 Donders Institute for Brain, Cognition and Behaviour, Radboud University, Nijmegen, The Netherlands; 3 Department of Psychology and Cognitive Science, University of Trento, Trento, Italy; 4 Psychology Program, Division of Science, New York University Abu Dhabi, Abu Dhabi, United Arab Emirates

**Keywords:** Psychology, Human behaviour

## Abstract

The world around us appears stable and continuous despite saccadic eye movements. This apparent visual stability is achieved by trans-saccadic perception leading at the behavioural level to preview effects: performance in processing a foveal stimulus is better if the stimulus remained unchanged (valid) compared to when it changed (invalid) during the saccade that brought it into focus. Trans-saccadic perception is known to predictively adapt to the statistics of the environment. Here, we asked whether the behavioural preview effect shows the same characteristics, employing a between-participants training design. Participants made saccades to faces which could change their orientation (upright/inverted) during the saccade. In addition, the post-saccadic face was slightly tilted and participants reported this tilt upon fixation. In a training phase, one group of participants conducted only invalid trials whereas another group conducted only valid trials. In a subsequent test phase with 50% valid and 50% invalid trials, we measured the preview effect. Invalid training reduced the preview effect. With a mixed-model analysis, we could show how this training effect gradually declines in the course of the test phase. These results show that the behavioural preview effect adapts to the statistics of the environment suggesting that it results from predictive processes.

## Introduction

We make about three saccadic eye-movements per second in everyday life. With these saccades, visual input changes drastically. We do not, however, notice these changes and we have the impression that the world around us is present in a stable and detailed fashion^1–3^. Attempting to explain this apparent discrepancy, past research revealed a number of interrelated features and feats of the visual system that contribute to the phenomenon of visual stability, such as saccadic suppression^4^, corollary discharge^5^, predictive remapping^6–8^, and trans-saccadic integration^9–11^. Among these explanations, a central idea is that trans-saccadic processing involves a form of predictive processing^3,12,13^. How exactly predictive processes contribute to stable visual perception is, however, not yet fully understood.

Considering the abundance of theories about predictive processing^14–19^, it is important to clarify what kind of effects can be meaningfully accommodated in predictive processing frameworks in the first place. We are aware that the term prediction, even if limited to perceptual processing, encompasses a wide range of phenomena and can be conceptualized in different ways. Here, we conceive of prediction as a type of processing that adapts on a rather short time scale—that is in the course of an experimental session of several minutes up to one hour—to certain statistical regularities concerning the frequency of events^20^ and we investigated whether the behavioural preview effect with faces qualifies as such a process. Alternatively, the behavioural preview effect could simply not be susceptible to statistical regularities at the temporal scale of an experimental session but rather depend only on the aspects of each single trial, due to, for example, spatiotopic neural adaptation or repetition suppression^19,21,22^.

The behavioural preview effect is closely linked to trans-saccadic perception. Every time we make a saccade, we focus the high-resolution part of the retina, the fovea, on a certain spot in the visual field. Perception at this new location is, however, influenced by what we could see less clearly at that location before the saccade. If a stimulus changes during a saccade (invalid preview), processing the post-saccadic stimulus is worse as reflected in worse behavioural performance compared to when the stimulus did not change (valid preview)^23–26^. Buonocore and colleagues^24^, for instance, found worse performance in a gender discrimination task with human faces in a condition where the face changed from a scrambled to an intact face across the saccade compared to a condition where it did not change, i.e. where the intact face was already shown before the saccade. Preview effects are particularly well established in the domain of reading research^27–30^.

Considering that the behavioural preview effect involves a match between pre-saccadic and post-saccadic processing, while also considering that trans-saccadic processing is at least to some extent predictive, the question arises whether the behavioural preview effect can be conceived of as the result of a predictive process. The idea here is that a change in visual input from before to after a saccade violates perceptual expectations^23^. Before a saccade is made, the visual system activates perceptual content which is likely to be encountered after the saccade. However, if not the expected input but something else is encountered (invalid preview), this new input triggers a prediction error and leads to worse and/or slower behavioural performance processing this new input. The basis for trans-saccadic predictions is presumably current peripheral visual input together with prior experience about how visual input changes from peripheral to foveal views. This idea is strongly supported by, for instance, Herwig and Schneider^12^ who showed that consistent trans-saccadic changes in the spatial frequency of vertical gratings during an acquisition phase affected judgments about the spatial frequency of peripherally viewed gratings in a following test phase^31^. Similarly, Valsecchi and Gegenfurtner^32^ provide evidence that peripherally viewed discs appear smaller (or larger) if they have been repeatedly replaced by a smaller (or larger) disc during a saccade directed towards them in the course of a training phase. These studies suggest that the visual system picks up on statistical regularities of how a stimulus changes from peripheral to foveal views leading eventually to altered peripheral perceptual experience. We wanted to know whether similar principles apply to the behavioural preview effect; in other words, we wanted to know whether the behavioural preview effect adapts to the statistical regularities of trans-saccadic changes. If that were the case, the behavioural preview effect could be considered as the result of predictive processing.

We have previously investigated the predictive nature of the preview effect with combined electroencephalography (EEG) and eye-tracking^23^. This study revealed several stages of pre- and post-saccadic processing which were differentially affected by statistical regularities in trans-saccadic changes. Whereas pre-saccadic stages were influenced by the frequency of trans-saccadic changes, early post-saccadic stages were largely unaffected. Apart from these neurophysiological findings, the statistical evidence for whether the *behavioural* preview effect resulted from predictive processing was rather unclear (p-value around 0.03 and a Bayes factor suggesting the opposite result)^23^. In this study, statistical regularities were manipulated with blocks of 66% valid compared to 33% valid trials. With the current study we want to shed more light on this issue.

To answer the question of whether the trans-saccadic behavioural preview effect reflects predictive processes, we conducted a para-foveal preview experiment and employed a between-participants training design. In our gaze-contingent experiment, a pre-saccadic perifoveal face stimulus could change (invalid preview) or maintain (valid preview) orientation (upright or inverted) during the eye movement directed towards it. The task was to discriminate a tiny tilt (left or right) of the post-saccadic face, also called the target face. One group of participants conducted a training phase with only valid trials during which the upright or inverted preview face remained the same across the saccade. The other group conducted a training phase with only invalid trials where the upright or inverted preview face always changed its orientation across the saccade. After the training phase, both groups of participants performed the same test phase with an equal frequency of valid and invalid trials. In this test phase, we measured the behavioural preview effect as the difference in post-saccadic face tilt discrimination performance between valid and invalid trials.

If the preview effect resulted from predictive processes, then the invalid training should result in a smaller preview effect compared to the valid training. The idea is that because of invalid training, trans-saccadic changes become more expected which increases performance in invalid conditions and reduces the difference in performance between valid and invalid trials. Moreover, if trans-saccadic perception flexibly adapts to the statistics of the environment, the expected influence of training is supposed to decay in the course of the test phase which contains the same number of valid and invalid trials for both training groups. We assessed this expected pattern of results by including trial number as a numeric predictor in a mixed model analysis. This setup provided the maximum temporal resolution to track changes in the behavioural preview effect across time, that is, at the single-trial level.

## Methods

### Participants

Data from 41 participants was collected. However, six participants performed with less than 60% average accuracy in the tilt discrimination task; therefore, they were excluded from the analysis. The remaining 35 participants had a mean age of 22.5 years, ranging from 18 to 41. Twenty-five of them were female, 29 right-handed, and 21 right-eye-dominant. All participants had normal or corrected-to-normal vision which was confirmed by an eyesight test using a Snellen chart. They received a monetary reimbursement for their participation and gave written informed consent. The study was approved by the Human Research Ethics Committee at the University of Trento and conducted in line with the Declaration of Helsinki.

### Apparatus and stimuli

The experiment was programmed in Matlab (The Mathworks Inc., Natick, MA, USA) with the Psychophysics toolbox^33,34^. We used 16 face stimuli and their phase-scrambled counterparts from one of our previous studies (Experiment 2 of Huber-Huber et al., 2019, originals from the Nottingham face data base, http://pics.stir.ac.uk/zips/nottingham.zip). As in the previous study, a circular mask with a diameter of 2.88° ensured that internal facial features, such as eyes, eyebrows, nose, and mouth, were equally well represented in each image while the head shape was occluded. Low-level statistics of the faces and their phase-scrambled counterparts were controlled by equating the luminance histograms of all stimuli to the average luminance histogram across all face stimuli with the *avgHist* and *histMatch* function of the SHINE toolbox^35^. Face images were presented at 8° eccentricity to the left of a 0.5° by 0.5° fixation cross.

For technical reasons, the data had to be collected in two different rooms with different computer monitors and the same eye-tracker model in different mounting setups. The data of the first 21 and the last four participants were recorded with a ViewPixx monitor with scanning LED backlight design (ViewPixx/EEG, VPX-VPX-2006A, by VPixx Technologies Inc., Saint-Bruno, Ontario, Canada), with a symmetric pixel rise and fall time of 1 ms specifically designed for vision research to replace cathode-ray tube monitors, running at 120 Hz screen refresh rate, the data of the other 16 participants were recorded with a cathode-ray tube monitor (Mitsubishi Diamond Pro 2070SB) at 85 Hz screen refresh rate (see below for implications on display timing). We consider both monitors equal enough in their display properties in order that any differences are irrelevant for the current research purpose. Both sets of participants were recorded with Eyelink 1000 eye-trackers (SR Research, Ontario, Canada). The eye-tracker for the first set was in desktop mount mode and the eye-tracker for the second set was in tower mount mode. For both eye-trackers we used default settings for saccade detection (velocity threshold 35°/s, acceleration threshold 9500°/s^2^). The eye-trackers were set to record the dominant eye which was determined by a hole-in-the-card test.

### Procedure and design

**Figure 1:**
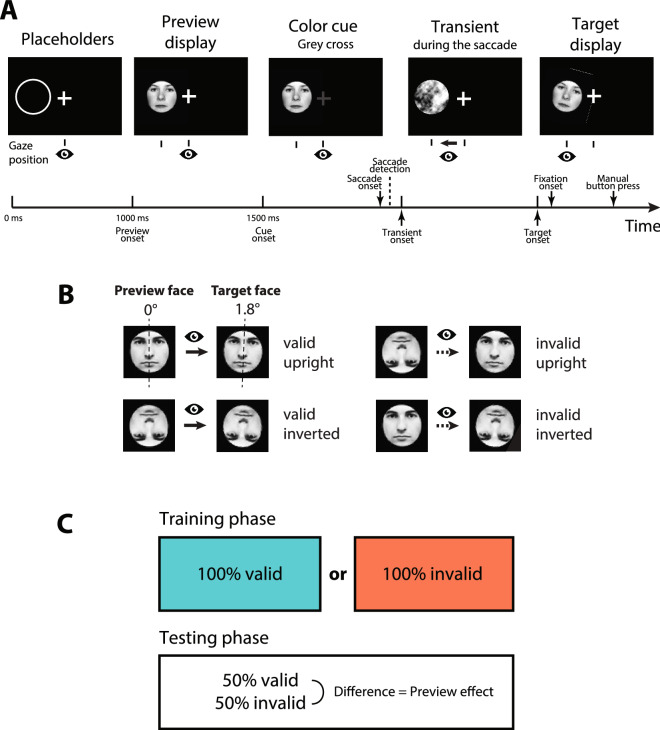
Trial procedure and experimental design. (**A**) Example of a valid preview trial with an upright target face. Stable gaze during the placeholder and preview periods made the fixation cross turn grey which cued the participant to look at the face. During the saccade, a visual transient equated low-level visual changes for valid and invalid trials. In most of the trials, the target face was presented before fixation onset. It had a small tilt which had to be reported by button press upon fixation. Stimuli, target face tilt and timeline are not drawn to scale. (**B**) All possible within-participant conditions of Target Orientation (upright, inverted) and Preview (valid, invalid). Only the target face was slightly tilted which had to be reported by manual button press. (**C**)Between-participants training setup. Each participant performed a training phase with either only valid or only invalid trials. After the training phase, the preview effect, i.e. difference in tilt discrimination performance between valid and invalid trials, was measured in a separate test phase with an equal amount of valid and invalid trials. (Face stimuli taken from the Nottingham face data base, http://pics.stir.ac.uk/zips/nottingham.zip).

Each trial started with a white fixation cross and a white placeholder ring of one pixel width presented on black background (illustrated in Fig. 1). After the eye tracker detected stable gaze for 1000 ms, an upright (0°) or inverted (180°) preview face was presented. After another 500 ms of stable gaze, the fixation cross turned grey prompting the participant to look at the preview face. An online saccade detection algorithm detected the start of the saccade defined as two subsequent gaze samples more than 0.18° apart on screen. This procedure led to satisfactory results in the past^23^. Detecting a saccade triggered the presentation of a transient for 16.7 ms, corresponding to two frames on the scanning LED backlight monitor running at 120 Hz screen refresh rate for the first set of 21 participants, or 11.8 ms, corresponding to one frame on the CRT monitor running at 85 Hz screen refresh rate for the remaining 16 participants (cf. Apparatus and stimuli). The transient was the phase-scrambled version of the preview face and served to balance the amount of trans-saccadic change in visual input between valid and invalid preview conditions^4,36^. After the transient, the same face image was presented again as target with the same (valid preview trial) or the opposite orientation (invalid preview trial). This design yielded the within-participant factors Preview (valid, invalid) and Target Orientation (upright, inverted). In addition to its overall orientation (upright or inverted) the target face exhibited a small tilt (1.8°) of the facial midline either to the left or to the right which had to be reported by manual button press on a USB computer keyboard. The small tilt rendered the target face in the valid preview condition not exactly the same as the preview face. This design, however, leads to clear preview effects^23^ and renders the effect even more interesting because the to-be-reported feature is yet absent in the preview stimulus and, still, the preview stimulus affects post-saccadic processing. Participants were instructed to respond as quickly and accurately as possible. If there was a mistake in the trial procedure, e.g. participants moved their eyes before the 500 ms preview period had ended, the trial was restarted. The experimenter monitored online gaze detection during the experiment and initiated recalibration whenever progress stalled.

The experiment consisted of two parts, first a training and then a test phase. The training phase contained either only valid or only invalid trials (100% valid or 100% invalid). This pure training phase was followed by a mixed test phase containing the same number of valid and invalid trials (50% valid and 50% invalid). For each block of trials, upright and inverted target faces were equally distributed across the experiment. Each participant was assigned to either the valid or the invalid training condition depending on their arrival to the experiment. We paid, however, attention to replacing participants with very low performance within their group in order to balance the number of participants across groups. Finally, 17 participants had trained with valid and 18 participants with invalid trials. That means, in addition to the two within-participant factors Preview (valid, invalid) and Target Orientation (upright, inverted) the design contained a between-participants factor training (valid, invalid). Within each cell of the design the 16 individual face images repeated equally often. Each participant performed in total 1024 trials, 512 of them in the training and 512 in the test phase. Trials were grouped in blocks of 32 with optional breaks in between. Within the training and within the test phase the order of trials was randomized. For one participant the experiment had to be restarted during the test phase and related to that reason an additional block of 32 test trials is available for this participant.

### Data analysis

We analysed manual response times, measured with respect to cue onset, and task errors of the test phase with mixed models (package lme4, version 1.1–21^37^ in R, version 3.6.1^38^). All data and analysis code to reproduce statistics and figures are provided online (see section “Data availability”). Mixed-models provide the advantage of working with continuous numeric predictors, such as trial number, which enables us to estimate not only the effects of the experimental conditions but also how these effects change across time. The computed models were based on individual trial data and contained the random factor participant and the following fixed factors: the two-level within-participant factors Preview (invalid–valid) and Target Orientation (inverted–upright) as well as the two-level between-participants factor Training (invalid–valid). The contrasts of these factors were coded as successive difference contrasts (MASS package in R), which provides the advantage that the intercept of the model corresponds to the grand mean and the effect estimates for the factors correspond to the differences between conditions given the mean across the levels of the other factors. This approach is comparable to traditional analysis of variance (Anova) procedures. To estimate the effect of change across time we included the numeric predictor trial number which was scaled (z-score) to be numerically in about the same range as the other factors, i.e. around − 1 to 1. We also centred this numeric predictor on trial number one of the test phase which means that the effects of other factors will be estimated for the first trial of the test phase. This first trial was exactly our time point of main interest. We wanted to know whether the training phase influenced the preview effect without confounding contributions from the test phase itself. Above all, these settings ensured that we finally also got to know how the preview effect then changed further in the course of the test phase.

In order to determine outlier trials, we filtered response times with a three median absolute deviation filter per participant, condition, and correctness of the response, which means that trials with a response time further away than three times the median absolute deviation from the participant-by-condition-by-correctness median were excluded. This approach is very robust and preferred over other approaches based e.g. on standard deviations^39^. To ensure not only proper task behaviour but also proper target face fixation, we further limited the analysis to trials where the saccade end point was within 2.16° from the centre of the face stimulus. These criteria resulted in 12,617 trials (70.6%) for the response time and 15,765 trials (87.8%) for the error rate analysis.

To correct for the usual skewness of the response time distribution, we took the inverse and multiplied it by − 1 to maintain the direction of effects. Note that the inverse transformation turns the effect of trial number from a linear effect into an effect of decay across time. Response times decrease to a greater extent in the beginning but not much anymore thereafter approaching an asymptote with increasing trial numbers. Values were back-transformed and converted to milliseconds for plotting. The analysis of response times was limited to trials with only correct responses. To model the continuous variable response times, we used a linear mixed model (command *lmer* of the lme4 package in R). In contrast, the binary variable task error was modelled with a multilevel logistic regression, i.e. a generalized linear model with a logit link function (command *glmer* of the lme4 package in R).

To avoid overestimation of the significance of effects, we included random slopes and aimed for the maximum model^40^. We paid close attention, however, that all specified parameters could actually be estimated based on the given dataset, that is, we checked whether a model was singular or not^41^. The maximum non-singular random slopes model, aka the maximum identified model, was compared to a null model without random slopes by means of a likelihood ratio test and Akaike’s Information Criterion (AIC) in order to see whether the random slopes did improve the fit to the data. This was always the case.

To determine the significance of fixed effects, we calculated 95% profile confidence intervals of the maximum identified model. We consider an effect as significant if its profile confidence interval excluded zero.

## Results

### Training changes the preview effect and the influence of training declines in the course of the test phase

**Figure 2:**
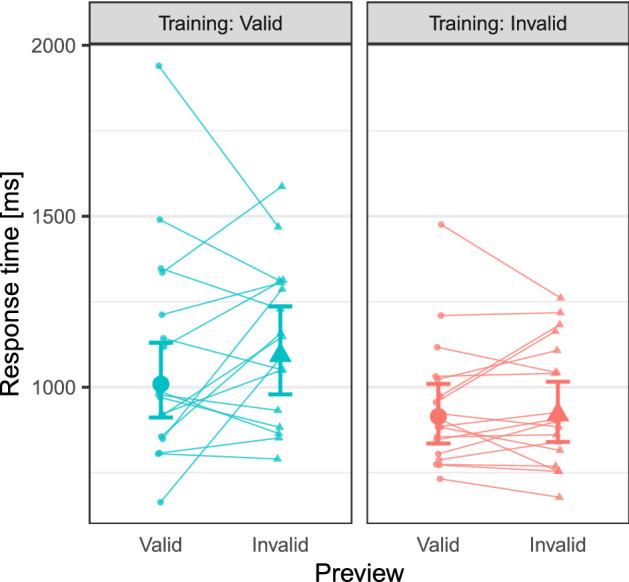
Estimated marginal means from the maximum identified model on response time data (Model 2). Individual participants' conditional modes are illustrated with smaller symbols and thin lines connecting the valid and invalid preview points. The preview effect, the difference between valid and invalid preview trials, depended on the training condition. In contrast to valid training (left side), there was no evidence for a preview effect with invalid training (right side, see also Models 2a and 2b below). Note that effect estimates were obtained for the first trial of the test phase. Error bars represent 95% asymptotic confidence intervals.

**Figure 3:**
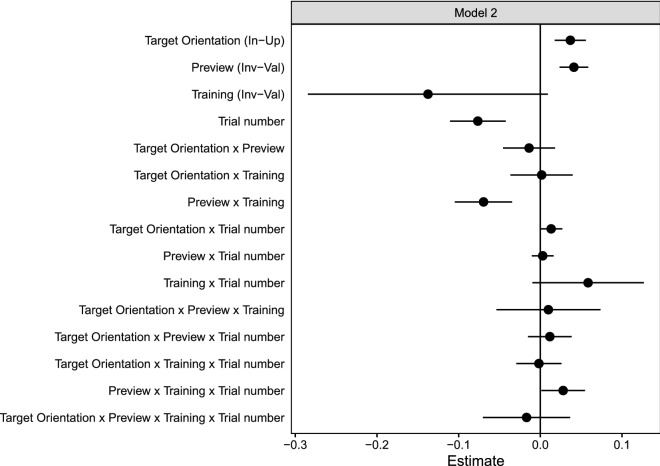
Fixed effect coefficients of the maximum identified linear mixed model on response times (Model 2). Error bars represent 95% profile confidence intervals. Effect contrasts for each factor are given in brackets next to the names of main effects on the y-axis. *In-Up* Inverted minus upright, *Inv-Val* Invalid minus valid. For example, a positive estimate for the Preview effect (invalid minus valid) means that responses were slower for invalid than for valid trials.

As can be seen from Fig. 2, the training manipulation had the expected effect. The valid training group showed a larger preview effect than the invalid training group, Preview × Training interaction beta = − 0.070, SE = 0.018, *t* = − 3.848, CI = [− 0.105, − 0.034]. Note, that we obtained this estimate for the time point right after the training phase, for the first trial of the test phase. The complete set of fixed effect coefficients of the maximum identified model is shown in Fig. 3 (for the model comparisons that led to this model, see Supplementary Table S1). Moreover, separate models for the two training groups confirmed that there was no evidence for a preview effect with invalid training, beta = 0.007, SE = 0.011, *t* = 0.607, CI = [− 0.015, 0.028] (model 2b, see Supplementary Table S2), but a significant preview effect with valid training, beta = 0.076, SE = 0.015, *t* = 5.154, CI = [0.047, 0.105] (model 2a, see Supplementary Table S3).

**Figure 4:**
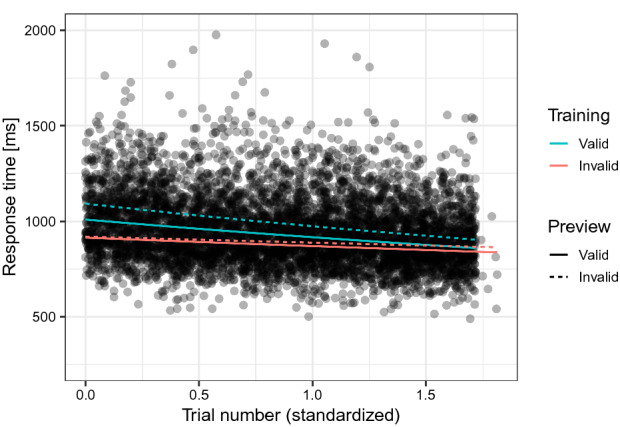
Response times showed an interaction of Training × Preview × Trial Number, which suggested that training with only valid trials resulted in a larger preview effect than training with only invalid trials particularly in the beginning of the test phase (Model 2). The preview effect then evolved in opposite directions for both training groups. Compared to the invalid training group, the preview effect in the valid training group declined. Dots represent a random sample of half of all individual data points. Trial number was standardized and centred on the first trial of the test phase.

Importantly, Fig. 3 revealed that the preview effect was not only influenced by training but that this influence also varied over time as indicated by a Preview × Training × Trial Number interaction, beta = 0.028, SE = 0.014, *t* = 2.051, CI = [0.001, 0.055]. This interaction is further illustrated in Fig. 4 where the preview effect corresponds to the difference between the solid (valid preview) and dashed (invalid preview) regression lines measured in the direction of the y-axis. As can be seen from this figure, the initial pattern of a preview effect for the valid training but not for the invalid training group (see also Fig. 2) converged and equalled out with time. In sum, this finding confirms that the preview effect was modulated by training and it adapted from trial to trial to the frequency of trans-saccadic changes.

Apart from the crucial and central Preview × Training × Trial Number interaction, the main effect of Target Orientation (inverted–upright) was significant, beta = 0.037, SE = 0.010, *t* = 3.749, CI = [0.018, 0.056]. Given its contrast of inverted face minus upright face, the positive beta of this effect indicated that participants were faster with upright than with inverted targets.

### Task errors do not confound the results in response times

**Figure 5:**
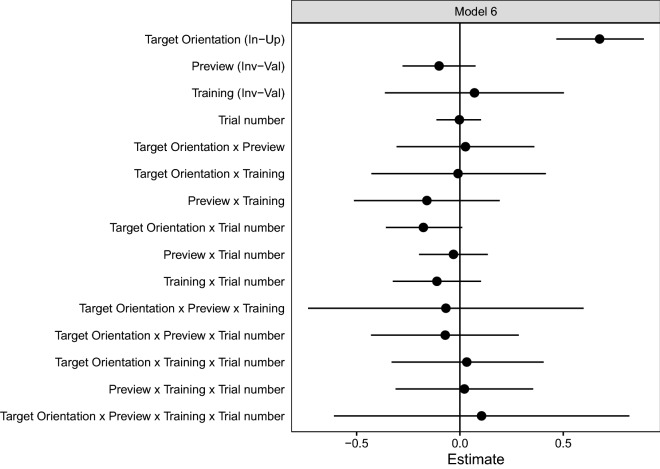
Fixed effect coefficients of the maximum identified generalized linear model on task errors (Model 6). Error bars represent 95% profile confidence intervals. Effect contrasts for each factor are given in brackets next to the names of main effects on the y-axis. *In-Up* Inverted minus upright, *Inv-Val* Invalid minus valid. For example, a positive estimate for the Target Orientation effect (inverted minus upright) means that there were more errors with inverted than with upright target faces.

In contrast to the response time analysis above, task errors did not show any particular pattern of results across time of the test phase in connection with Training or Preview. Importantly, this result rules out any speed-accuracy trade-offs. The corresponding fixed effect coefficients of the maximum identified model are illustrated in Fig. 5 (for the model comparisons that led to this model, see Supplementary Table S4). As can be seen from this figure, we only found a significant main effect of Target Orientation (inverted–upright), beta = 0.676, CI = [0.467, 0.890], showing that tilt discrimination performance was better with upright than with inverted faces, which is in line with the corresponding effect in response times.

## Discussion

In the present study, we investigated whether the behavioural preview effect can be conceived of as the result of a predictive process. In a between-participants training design, we manipulated the frequency of trans-saccadic changes with only valid or invalid trials in the training phase and an equal number of valid and invalid trials in the subsequent test phase. Mixed model analyses of response times and task errors with trial number as numeric predictor showed that the preview effect in response times, first, is affected by training. In line with our hypothesis, the invalid training group did not provide any evidence for a preview effect at the beginning of the test phase. In contrast, valid training showed a clear preview effect. The second important result from our study is that this initial difference in the preview effect due to training gradually converged and equalled during the test phase. Towards the end of the test phase the two groups showed more similar preview effects than at the beginning of the test phase. This result confirms the idea that the behavioural preview effect reflects a form of predictive processing that adapts flexibly on a small time scale, in our case a period measured in minutes, to the statistics of the environment in terms of frequency of events.

Our findings corroborate and extend previous research on how trans-saccadic perception adapts to the statistics of the environment^12,31,32^. Similar to, for instance, Herwig and Schneider^12^, we found that repeated exposure to trans-saccadic changes during an acquisition phase influences trans-saccadic processing in a subsequent test phase. Whereas Herwig and Schneider showed that peripherally viewed objects appeared to have a higher spatial frequency if they always increased in spatial frequency during the saccade directed towards it, we provide evidence for the behavioural consequences of repeated exposure to trans-saccadic changes. Moreover, our results extend previous research about simple visual features like spatial frequency^12^, shape^31^, and size^32^ to more complex visual input like human faces.

Reconsidering the results from previous research on the trans-saccadic preview effect with faces^23^, we might even speculate about the neural sources of the behavioural preview effect. Our previous EEG results suggested that in particular the pre-saccadic part of trans-saccadic processing is susceptible to the frequency of events and the statistics of trans-saccadic changes. Considering this finding, the training manipulation of the current experiment might have in particular affected this pre-saccadic stage. Possibly, the invalid training changed pre-saccadic processing in a way that finally led to a reduced influence of the preview face on post-saccadic target face processing.

Besides our main conclusion that the behavioural preview effect reflects a predictive process that flexibly adapts to the statistics of the environment^20^, there is a second inter-related respect in which trans-saccadic perception can be considered as predictive. In this second respect, trans-saccadic prediction means that the visual system instantiates in some way, already before the saccade, the expected post-saccadic input^12^. This view would imply a somewhat stricter hypothesis in the sense that the preview effect would not only have to be affected by training, but should even be reversed after invalid compared to after valid training. In this view, invalid training should make the visual system instantiate already before the saccade the face orientation opposite to the one actually present in the periphery, which would further imply better performance in the invalid than in the valid condition, that is, a reversed preview effect. Our results do not contradict this stricter interpretation of trans-saccadic predictions, however, our experimental design can also in principle not corroborate it. We did not find a reversed preview effect and it is in theory possible that more extensive training would have led to a reversed preview effect. Alternatively, prediction and learning may have bounds making trans-saccadic prediction in this second respect even impossible with large changes in the stimulus like in our experiment. Previous studies looking for learning across trials have typically used relatively small transformations. Similarly, it is easier to adapt saccadic amplitude if the displacement of the landing point is relatively small on any given trial^42^. Saccadic suppression of displacement occurs only when the displacement during the saccade is relatively small^43^. In our case, a 180 degree rotation might be too much of a change to create an association and evoke learning, or it might create a consistent error signal that eliminates statistical learning. However, these arguments apply whenever no reversed preview effect is found. Thus, to investigate this stricter interpretation, other experimental designs and perhaps other research methods are required (e.g. decoding of pre-saccadic neural activity^44^).

Apart from the crucial results about trans-saccadic perception, this dataset also shows a very clear face inversion effect in line with previous findings^23^. Tilt discrimination of upright target faces was better than that of inverted faces which is possibly related to the impaired recognition of inverted compared to upright faces^45^. This effect, and the Target Orientation factor in the first place are, however, not relevant to trans-saccadic perception but a simple by-product of balancing upright and inverted faces across preview and target displays.

More generally, one interesting implication of the present results is that the proportion of valid trials within an experimental design may greatly affect the degree to which pre-saccadic visual input influences post-saccadic visual processing. The proportion of *valid preview* has varied extensively across the numerous studies of trans-saccadic perception conducted in various laboratories thus far. In some experiments, valid trials were the majority within a block or even blocked together. Our results suggest this leads to the highest likelihood of finding a trans-saccadic effect. In contrast, many other experiments have been designed in a way that valid predictions about the post-saccadic stimulus are licensed only on a third or a fourth of trials, or even less^9,10,24,25,28,46–50^. Typically, there is one *valid* match in spatial coordinators or in feature space, combined with two or more invalid conditions in which the location or visual features of the stimulus changes during the saccade in a way that is very unlikely in the real world. The current results suggest that experimental designs used in the past may have underestimated the magnitude, or even presence, of trans-saccadic prediction effects. Furthermore, our results raise the question of whether spatiotopic trans-saccadic effects may be more strongly influenced by the number of invalid conditions than retinotopic effects, because retinotopic effects are per definition invalid in at least their spatial aspect given our usually spatiotopically stable visual environment. We recommend for future research to be aware of this issue and suggest to design experimental protocols accordingly. For instance, we expect that randomizing trials across an equal number of spatiotopic and retinotopic conditions prevents any systematic validity effects. One possible strategy would be to include a proportion manipulation, such as for example within blocks, in order to always include blocks in which spatiotopic position is matched on a majority of trials**.** However, further systematic investigations of this issue are required before clear recommendations can be given.

In sum, the present study demonstrates the prominent influence of statistical regularities on trans-saccadic perception and it suggests that the behavioural preview effect reflects predictive processes that flexibly adapt to the statistics of the environment.

## Supplementary Information

Supplementary Information 1.

Supplementary Information 2.

Supplementary Information 3.

Supplementary Information 4.

## Data Availability

The raw data can be obtained from https://doi.org/10.17605/OSF.IO/TY69K. Analysis code is available at GitHub https://github.com/chsquare/beh_pre_eff with the registered digital object identifier https://doi.org/10.5281/zenodo.3746057. This code contains an R markdown file to run the complete analysis and generate a report rendered in html format with all statistics and figures of the article and further supplementary information. A ready-to-use version of the html file is included as well in the code repository https://github.com/chsquare/beh_pre_eff and on https://doi.org/10.17605/OSF.IO/TY69K from where it can be downloaded separately and opened directly with any web browser.
