# Supplementary material for: The behavioural preview effect with faces is susceptible to statistical regularities: Evidence for predictive processing across the saccade
Source: Sci Rep. 2021 Jan 13;11:942. doi: 10.1038/s41598-020-79957-w (PMC7806959; doi:10.1038/s41598-020-79957-w)
Supplement: Supplementary file 1 — Supplementary Information 1. [file 41598_2020_79957_MOESM1_ESM.html]

|  |  |  |  |  |
| --- | --- | --- | --- | --- |
|  | | | | |
|  | *Dependent variable:* | | | |
|  | -1 / Response time [sec] | | | |
|  |  | | | |
|  | *Fitting method:* | | | |
|  | REML | REML | REML | REML |  |
|  |  | | | |
|  | (1) | (2) | (3) | (4) |
|  | | | | | |
| **Random effects variances** |  | | | | |
| *Participant* |  | | | | |
| (Intercept) | 0.039 | 0.049 | 0.049 | 0.049 |
| Target Orientation (In-Up) |  | 0.002 | 0.002 | 0.002 |
| Preview (Inv-Val) |  | 0.001 | 0.001 | 0.001 |
| Trial number |  | 0.010 | 0.010 | 0.010 |
| Target Orientation x Preview |  | 0.003 | 0.003 | 0.003 |
| Target Orientation x Trial number |  | 0.0001 | 0.0001 | 0.0001 |
| Preview x Trial number |  |  | 0 | 0 |
| Target Orientation x Preview x Trial number |  |  |  | 0 |
|  |  | | | | |
| Residual Variance | 0.039 | 0.036 | 0.036 | 0.036 |
|  | | | | | |
| **Fixed effects** |  | | | |
| Target Orientation (In-Up) | 0.033 | 0.037 | 0.037 | 0.037 |
|  | (0.007) | (0.010) | (0.010) | (0.010) |
|  | t = 4.675 | t = 3.749 | t = 3.749 | t = 3.749 |
|  |  |  |  |  |
| Preview (Inv-Val) | 0.044 | 0.041 | 0.041 | 0.041 |
|  | (0.007) | (0.009) | (0.009) | (0.009) |
|  | t = 6.143 | t = 4.549 | t = 4.549 | t = 4.549 |
|  |  |  |  |  |
| Training (Inv-Val) | -0.144 | -0.138 | -0.138 | -0.138 |
|  | (0.068) | (0.075) | (0.075) | (0.075) |
|  | t = -2.128 | t = -1.831 | t = -1.831 | t = -1.831 |
|  |  |  |  |  |
| Trial number | -0.074 | -0.077 | -0.077 | -0.077 |
|  | (0.004) | (0.017) | (0.017) | (0.017) |
|  | t = -20.736 | t = -4.388 | t = -4.388 | t = -4.388 |
|  |  |  |  |  |
| Target Orientation x Preview | -0.010 | -0.014 | -0.014 | -0.014 |
|  | (0.014) | (0.016) | (0.016) | (0.016) |
|  | t = -0.729 | t = -0.841 | t = -0.841 | t = -0.841 |
|  |  |  |  |  |
| Target Orientation x Training | 0.010 | 0.002 | 0.002 | 0.002 |
|  | (0.014) | (0.020) | (0.020) | (0.020) |
|  | t = 0.696 | t = 0.083 | t = 0.083 | t = 0.083 |
|  |  |  |  |  |
| Preview x Training | -0.074 | -0.070 | -0.070 | -0.070 |
|  | (0.014) | (0.018) | (0.018) | (0.018) |
|  | t = -5.242 | t = -3.848 | t = -3.848 | t = -3.848 |
|  |  |  |  |  |
| Target Orientation x Trial number | 0.016 | 0.013 | 0.013 | 0.013 |
|  | (0.007) | (0.007) | (0.007) | (0.007) |
|  | t = 2.269 | t = 1.866 | t = 1.866 | t = 1.866 |
|  |  |  |  |  |
| Preview x Trial number | 0.001 | 0.003 | 0.003 | 0.003 |
|  | (0.007) | (0.007) | (0.007) | (0.007) |
|  | t = 0.079 | t = 0.425 | t = 0.425 | t = 0.425 |
|  |  |  |  |  |
| Training x Trial number | 0.065 | 0.058 | 0.058 | 0.058 |
|  | (0.007) | (0.035) | (0.035) | (0.035) |
|  | t = 9.151 | t = 1.676 | t = 1.676 | t = 1.676 |
|  |  |  |  |  |
| Target Orientation x Preview x Training | 0.015 | 0.010 | 0.010 | 0.010 |
|  | (0.028) | (0.033) | (0.033) | (0.033) |
|  | t = 0.522 | t = 0.300 | t = 0.300 | t = 0.300 |
|  |  |  |  |  |
| Target Orientation x Preview x Trial number | 0.007 | 0.012 | 0.012 | 0.012 |
|  | (0.014) | (0.014) | (0.014) | (0.014) |
|  | t = 0.486 | t = 0.854 | t = 0.854 | t = 0.854 |
|  |  |  |  |  |
| Target Orientation x Training x Trial number | -0.007 | -0.002 | -0.002 | -0.002 |
|  | (0.014) | (0.014) | (0.014) | (0.014) |
|  | t = -0.469 | t = -0.122 | t = -0.122 | t = -0.122 |
|  |  |  |  |  |
| Preview x Training x Trial number | 0.033 | 0.028 | 0.028 | 0.028 |
|  | (0.014) | (0.014) | (0.014) | (0.014) |
|  | t = 2.359 | t = 2.051 | t = 2.051 | t = 2.051 |
|  |  |  |  |  |
| Target Orientation x Preview x Training x Trial number | -0.015 | -0.017 | -0.017 | -0.017 |
|  | (0.028) | (0.027) | (0.027) | (0.027) |
|  | t = -0.541 | t = -0.618 | t = -0.618 | t = -0.618 |
|  |  |  |  |  |
| Constant | -1.025 | -1.022 | -1.022 | -1.022 |
|  | (0.034) | (0.038) | (0.038) | (0.038) |
|  | t = -30.344 | t = -27.213 | t = -27.213 | t = -27.213 |
|  |  |  |  |  |
|  | | | | |
| Observations | 12,671 | 12,671 | 12,671 | 12,671 |
| AICc | -4852.342 | -5702.931 | -5700.924 | -5698.916 |
| Log Likelihood | 2444.198 | 2874.509 | 2874.509 | 2874.509 |
| Deviance | -4888.396 | -5749.019 | -5749.019 | -5749.019 |
| Df | 18 | 23 | 24 | 25 |
| *χ2* |  | 860.623 | 0 | 0 |
| *χ2* Df |  | 5 | 1 | 1 |
| *p* |  | < .001 | 1.000 | 1.000 |
| Model is singular |  |  | † | † |
|  | | | | |
|  | | | | |
