# Supplementary material for: The behavioural preview effect with faces is susceptible to statistical regularities: Evidence for predictive processing across the saccade
Source: Sci Rep. 2021 Jan 13;11:942. doi: 10.1038/s41598-020-79957-w (PMC7806959; doi:10.1038/s41598-020-79957-w)
Supplement: Supplementary file 2 — Supplementary Information 2. [file 41598_2020_79957_MOESM2_ESM.html]

**Supplementary Table S2. Fixed effects of Model 2a, the maximum identified model on response times of the valid training group. Estimate, standard error, t-value, and lower/upper limit of 95% profile confidence intervals.**

|  | | | | | |
| Parameter | Estimate | Std. Error | t value | 2.5 % | 97.5 % |
|  | | | | | |
| ((Intercept)) | -0.953 | 0.056 | -17.112 | -1.065 | -0.841 |
| Target Orientation (In-Up) | 0.036 | 0.013 | 2.799 | 0.011 | 0.061 |
| Preview (Inv-Val) | 0.076 | 0.015 | 5.154 | 0.047 | 0.105 |
| Trial number | -0.106 | 0.030 | -3.525 | -0.166 | -0.045 |
| Target Orientation x Preview | -0.019 | 0.024 | -0.800 | -0.067 | 0.028 |
| Target Orientation x Trial number | 0.014 | 0.009 | 1.524 | -0.004 | 0.032 |
| Preview x Trial number | -0.011 | 0.009 | -1.187 | -0.029 | 0.007 |
| Target Orientation x Preview x Trial number | 0.021 | 0.019 | 1.124 | -0.016 | 0.057 |
|  | | | | | |
