# Supplementary material for: The behavioural preview effect with faces is susceptible to statistical regularities: Evidence for predictive processing across the saccade
Source: Sci Rep. 2021 Jan 13;11:942. doi: 10.1038/s41598-020-79957-w (PMC7806959; doi:10.1038/s41598-020-79957-w)
Supplement: Supplementary file 3 — Supplementary Information 3. [file 41598_2020_79957_MOESM3_ESM.html]

**Supplementary Table S3. Fixed effects of Model 2b, the maximum identified model on response times of the invalid training group. Estimate, standard error, t-value, and lower/upper limit of 95% profile confidence intervals.**

|  | | | | | |
| Parameter | Estimate | Std. Error | t value | 2.5 % | 97.5 % |
|  | | | | | |
| ((Intercept)) | -1.091 | 0.051 | -21.520 | -1.193 | -0.989 |
| Target Orientation (In-Up) | 0.038 | 0.015 | 2.563 | 0.009 | 0.067 |
| Preview (Inv-Val) | 0.007 | 0.011 | 0.607 | -0.015 | 0.028 |
| Trial number | -0.047 | 0.019 | -2.535 | -0.084 | -0.010 |
| Target Orientation x Preview | -0.007 | 0.022 | -0.316 | -0.051 | 0.036 |
| Target Orientation x Trial number | 0.012 | 0.011 | 1.053 | -0.011 | 0.034 |
| Preview x Trial number | 0.017 | 0.010 | 1.676 | -0.003 | 0.036 |
| Target Orientation x Preview x Trial number | 0.002 | 0.020 | 0.118 | -0.037 | 0.041 |
|  | | | | | |
