# Supplementary material for: The behavioural preview effect with faces is susceptible to statistical regularities: Evidence for predictive processing across the saccade
Source: Sci Rep. 2021 Jan 13;11:942. doi: 10.1038/s41598-020-79957-w (PMC7806959; doi:10.1038/s41598-020-79957-w)
Supplement: Supplementary file 4 — Supplementary Information 4. [file 41598_2020_79957_MOESM4_ESM.html]

|  |  |  |  |  |
| --- | --- | --- | --- | --- |
|  | | | | |
|  | *Dependent variable:* | | | |
|  | Task error (log odds) | | | |
|  |  | | | |
|  | (5) | (6) | (7) | (8) |
|  | | | | |
| **Random effects variances** |  | | | |
| *Participant* |  | | | |
| (Intercept) | 0.344 | 0.340 | 0.340 | 0.340 |
| Target Orientation (In-Up) |  | 0.134 | 0.134 | 0.134 |
| Preview (Inv-Val) |  | 0.027 | 0.027 | 0.027 |
| Trial number |  | 0.032 | 0.032 | 0.032 |
| Target Orientation x Trial number |  | 0.043 | 0.043 | 0.043 |
| Target Orientation x Preview x Trial number |  | 0.136 | 0.136 | 0.136 |
| Preview x Trial number |  |  | 0 | 0 |
| Target Orientation x Preview |  |  |  | 0 |
|  |  | | | |
| Residual Variance | 1 | 1 | 1 | 1 |
|  | | | | |
| **Fixed effects** |  | | | |
| Target Orientation (In-Up) | 0.655 | 0.676 | 0.676 | 0.676 |
|  | (0.085) | (0.107) | (0.107) | (0.107) |
|  | t = 7.725 | t = 6.331 | t = 6.331 | t = 6.331 |
|  |  |  |  |  |
| Preview (Inv-Val) | -0.107 | -0.101 | -0.101 | -0.101 |
|  | (0.085) | (0.090) | (0.090) | (0.090) |
|  | t = -1.265 | t = -1.125 | t = -1.125 | t = -1.125 |
|  |  |  |  |  |
| Training (Inv-Val) | 0.082 | 0.070 | 0.070 | 0.070 |
|  | (0.216) | (0.216) | (0.216) | (0.216) |
|  | t = 0.381 | t = 0.326 | t = 0.326 | t = 0.326 |
|  |  |  |  |  |
| Trial number | 0.020 | -0.003 | -0.003 | -0.003 |
|  | (0.042) | (0.054) | (0.054) | (0.054) |
|  | t = 0.463 | t = -0.048 | t = -0.048 | t = -0.048 |
|  |  |  |  |  |
| Target Orientation x Preview | 0.028 | 0.027 | 0.027 | 0.027 |
|  | (0.169) | (0.170) | (0.170) | (0.170) |
|  | t = 0.166 | t = 0.158 | t = 0.158 | t = 0.158 |
|  |  |  |  |  |
| Target Orientation x Training | -0.045 | -0.009 | -0.009 | -0.009 |
|  | (0.170) | (0.213) | (0.213) | (0.213) |
|  | t = -0.265 | t = -0.043 | t = -0.043 | t = -0.043 |
|  |  |  |  |  |
| Preview x Training | -0.156 | -0.160 | -0.160 | -0.160 |
|  | (0.169) | (0.179) | (0.179) | (0.179) |
|  | t = -0.921 | t = -0.892 | t = -0.892 | t = -0.892 |
|  |  |  |  |  |
| Target Orientation x Trial number | -0.199 | -0.177 | -0.177 | -0.177 |
|  | (0.084) | (0.093) | (0.093) | (0.093) |
|  | t = -2.363 | t = -1.893 | t = -1.893 | t = -1.893 |
|  |  |  |  |  |
| Preview x Trial number | -0.023 | -0.031 | -0.031 | -0.031 |
|  | (0.084) | (0.085) | (0.085) | (0.085) |
|  | t = -0.267 | t = -0.370 | t = -0.370 | t = -0.370 |
|  |  |  |  |  |
| Training x Trial number | -0.115 | -0.111 | -0.111 | -0.111 |
|  | (0.084) | (0.106) | (0.106) | (0.106) |
|  | t = -1.363 | t = -1.053 | t = -1.053 | t = -1.053 |
|  |  |  |  |  |
| Target Orientation x Preview x Training | -0.071 | -0.068 | -0.068 | -0.068 |
|  | (0.339) | (0.340) | (0.340) | (0.340) |
|  | t = -0.210 | t = -0.200 | t = -0.200 | t = -0.200 |
|  |  |  |  |  |
| Target Orientation x Preview x Trial number | -0.057 | -0.071 | -0.071 | -0.071 |
|  | (0.169) | (0.182) | (0.182) | (0.182) |
|  | t = -0.338 | t = -0.391 | t = -0.391 | t = -0.391 |
|  |  |  |  |  |
| Target Orientation x Training x Trial number | 0.006 | 0.033 | 0.033 | 0.033 |
|  | (0.169) | (0.186) | (0.186) | (0.186) |
|  | t = 0.034 | t = 0.178 | t = 0.178 | t = 0.178 |
|  |  |  |  |  |
| Preview x Training x Trial number | 0.027 | 0.022 | 0.022 | 0.022 |
|  | (0.169) | (0.170) | (0.170) | (0.170) |
|  | t = 0.160 | t = 0.127 | t = 0.127 | t = 0.127 |
|  |  |  |  |  |
| Target Orientation x Preview x Training x Trial number | 0.088 | 0.105 | 0.105 | 0.105 |
|  | (0.337) | (0.363) | (0.363) | (0.363) |
|  | t = 0.261 | t = 0.288 | t = 0.288 | t = 0.288 |
|  |  |  |  |  |
| Constant | -1.538 | -1.543 | -1.543 | -1.543 |
|  | (0.108) | (0.108) | (0.108) | (0.108) |
|  | t = -14.216 | t = -14.257 | t = -14.257 | t = -14.257 |
|  |  |  |  |  |
|  | | | | |
| Observations | 15,765 | 15,765 | 15,765 | 15,765 |
| AICc | 14817.779 | 14775.659 | 14777.665 | 14779.671 |
| Log Likelihood | -7391.87 | -7365.797 | -7365.797 | -7365.797 |
| Deviance | 14783.741 | 14731.595 | 14731.595 | 14731.595 |
| Df | 17 | 22 | 23 | 24 |
| *χ2* |  | 52.146 | 0 | 0 |
| *χ2* Df |  | 5 | 1 | 1 |
| *p* |  | < .001 | 1.000 | 1.000 |
| Model is singular |  |  | † | † |
|  | | | | |
|  | | | | |
